# P53 and Expression of Immunological Markers May Identify Early Stage Thyroid Tumors

**DOI:** 10.1155/2013/846584

**Published:** 2013-09-19

**Authors:** Marjory Alana Marcello, Elaine Cristina Morari, Lucas Leite Cunha, Aline Carolina De Nadai Silva, Dirce Maria Carraro, André Lopes Carvalho, Fernando Augusto Soares, José Vassallo, Laura Sterian Ward

**Affiliations:** ^1^Laboratory of Cancer Molecular Genetics, Faculty of Medical Sciences (FCM), University of Campinas (Unicamp), Rua Tessalia Vieira de Camargo 126, Cidade Universitaria Zeferino Vaz, Campinas 13083-887, SP, Brazil; ^2^Department of Biological Sciences and Health, State University of Roraima (UERR), Rua Sete de Setembro 231, Boa Vista, RR, Brazil; ^3^Laboratory of Genomics and Molecular Biology, CIPE, AC Camargo Hospital, Rua Tagua 440, 1**º** andar, São Paulo, SP, Brazil; ^4^Department of Head and Neck Surgery, Barretos Cancer Hospital, Avenida Antenor Duarte Villela 1331, Barretos, SP, Brazil; ^5^Department of Pathology, AC Camargo Cancer Center, Rua Prof. Antônio Prudente 109, 1**º** andar, Anatomia Patológica, São Paulo, SP, Brazil; ^6^Laboratory of Investigative and Molecular Pathology, Faculty of Medical Sciences (FCM), University of Campinas (Unicamp), Rua Tessalia Vieira de Camargo 126, Campinas, São Paulo, Brazil

## Abstract

*Background*. Besides its major role in cell proliferation, DNA repair, and apoptosis, functional p53 protein is involved in the induction of antitumor cytotoxic-T-cell activity against carcinoma cells. We aimed to investigate p53 and immune cell markers utility as diagnostic and prognostic markers of differentiated thyroid cancer (DTC). *Methods*. ACIS-III system was used to evaluate p53 and immune cell markers including tumor-associated macrophages (TAM); CD68 and tumor-infiltrating lymphocytes (TIL) subsets such as CD3, CD4, CD8, and CD20 in 206 thyroid carcinomas, 105 benign nodules, and 18 normal tissues. Also, *TP53* was sequenced in 78 out of 164 patients with papillary thyroid carcinoma. *Results*. P53 expression was observed more frequently in malignant than in benign lesions (*P* < 0.0001) and helped discriminate follicular patterned lesions. In addition, p53 was more frequent in smaller (*P* = 0.0015), unique tumors (*P* = 0.0286), with thyroiditis (*P* = 0.0486) and without metastasis at diagnosis (*P* = 0.0201). TAM was more frequent in P53 negative tumors (*P* = 0.002). Infiltration of CD8+ TIL was found in 61.7% of P53 positive and 25.6% of P53 negative DTC (*P* < 0.001). *Conclusions*. We suggest that p53 and CD8+ TIL immune profile analysis might be useful in DTC.

## 1. Introduction

The incidence of cancer has grown around the world during the last years mainly due to the large increase in the number of cases of small lesions detected because the aging population is obtaining better access to health care and sensitive diagnostic tools [1]. Although there are lines of evidence that a large number of these small tumors would never become clinically important [2], part of these lesions evolve, and despite the general good prognosis of differentiated thyroid cancer, up to 10–15% of patients have an aggressive form of the disease, which will contribute to the 1,850 deaths from thyroid cancer estimated to occur in 2013 in the USA [3]. 

Two major problems haunt the physicians who need to delineate the best management strategy for their patients with thyroid nodule: the first one is the indeterminate lesion. In fact, 15–30% of thyroid nodules evaluated by fine-needle aspiration cytology are not clearly benign or malignant [4]. A second critical issue is the identification of the most aggressive cases, especially among papillary thyroid cancers (PTCs), which would benefit from more aggressive therapy and followup, sparing the vast majority of thyroid tumors of invasive costly procedures [5].

A series of molecular markers that may improve the diagnostic and prognostic scoring systems currently used in these situations has been recently described; however, unfortunately, most of these systems still lacking accuracy are costly and/or difficult to implement in a clinical routine center [6, 7]. Hence, there is an imperative need for further developments in the approach to thyroid nodule, aiming to allow greater individualization of patient care. 

*TP53 *gene exerts a major role in the negative control of cell proliferation and in controlling signaling cascades important in DNA repair and apoptosis [8]. Our group and others have previously reported that the inheritance of* TP53* variants might be related to the risk of thyroid tumor development [9, 10], and there is data suggesting that *TP53 *mutations may play an important role in the malignant transformation of thyroid cells and thyroid tumor progression [11]. Because some studies have found *TP53 *mutations in poorly differentiated and undifferentiated thyroid carcinomas but not in benign tumors, and only in a few well-differentiated thyroid cancers, it has been suggested that mutational inactivation of *TP53* occurs at a late stage of tumor progression, contributing to the development of metastatic forms [12]. Interestingly, there is a low rate of loss of heterozygosity in chromosome 17p of differentiated thyroid tumors [13]. In addition, the status of *TP53 *gene product, either wild-type or mutant p53 nuclear protein, has also been linked to tumor chemosensitivity, radiosensitivity, prognosis, and antitumor immune response in many human cancer types [14–16].

In this study, we aimed to expand our understanding on *TP53* and its product alterations, as well as the associated clinicopathological features, to further investigate p53 use as a diagnostic and/or prognostic marker in DTC patients. In addition, we investigated the putative correlation between p53 expression and presence of tumor infiltrating lymphocytes (TIL) and tumor-associated macrophages (TAM).

## 2. Subjects and Methods

### 2.1. Patients

This study was approved by the Research Ethics Committee of the institutions involved. We investigated 311 patients whose tissue samples were maintained in the tissue bank of the Cancer Hospital AC Camargo, São Paulo, Brazil, summarized in Table 1. Two hundred six patients were diagnosed with thyroid carcinoma: 164 with PTC (106 with the classic form (CPTC); 54 with follicular variant (FVPTC); and 04 with tall cell variant (TCVPTC)), and 42 with follicular thyroid carcinomas (FTC). Malignant thyroid tissues collected at surgery immediately snap-frozen in liquid nitrogen and stored at −80°C for molecular studies were obtained from 78 of the patients. In addition, we obtained 105 benign thyroid tissues, including 50 nodular goiters (G) and 55 follicular adenomas (FA). We also obtained 18 normal thyroid tissues (NT) from the contralateral lobe of patients who presented a solitary follicular adenoma. Patients' demographic, laboratory, image, and clinical information were obtained from their charts. 

All tumors were carefully and independently reviewed by two experienced pathologists (JV and FAS) for diagnostic confirmation, and cases presenting conflicting results or areas of differentiation were excluded. Formalin-fixed, paraffin-embedded tissues were examined aiming to select the most representative areas designed to build a tissue microarray (TMA, Beecher Instruments, Silver Springs, MD, USA) for immunohistochemical detection of p53 and immune cell infiltration. We acquired four tumor tissue cores from each case. Two spots were chosen from representative areas of the lesion presenting important leukocyte infiltration, whereas two other spots were chosen from areas free of leukocytes.

P53 protein immunohistochemical expression was correlated with clinicopathological parameters, such as age, gender, ethnic group, tumor size, multifocality, extrathyroidal invasion, presence of lymph node metastases, and outcome (recurrence/metastasis-free survival), as summarized in Table 2. 

Patients were followed with periodic whole-body scans and serum TSH and thyroglobulin (Tg) measurements, according to a standard protocol based on current international management guidelines that included X-ray, ultrasonography, computed tomography scan, and other eventual procedures to detect distant metastasis for a period of 12–298 months (53.8 ± 41 months) [17]. Patients presenting or with suspicion of high nonstimulated serum Tg levels (>2 mg/dL) were submitted to a thorough image search. We defined tumors as recurrent and/or presenting long distance when patients showed serum Tg levels > 2 mg/dL and/or the above mentioned image scans were positive for metastasis. Thyroid cancer patients were classified as free of disease in 142 cases (85%) and with recurrence in 25 cases (15%, including 10 dead from disease). Thirty-nine patients could not be classified into any of these two groups and were further excluded from any analysis involving outcome. Aggressiveness at diagnosis was ascertained using American Joint Committee on Cancer (AJCC) TNM system for differentiated thyroid carcinoma [17]. 

### 2.2. Immunohistochemical (IHC) Detection of p53 and Infiltration of Immune Cells

Five micrometer sections of the TMA were placed on electrically charged slides, deparaffinized and rehydrated in decreasing concentrations of alcohol. The endogenous peroxide activity was blocked with H_2_O_2_ for 15 min. All tissue sections were subjected to heat-induced antigen retrieval, using 10% citrate buffer (10 mM, pH 6.0) in a steamer (90°C for 30 minutes). Tissue sections were then incubated overnight at 6°C, using monoclonal mouse anti-human p53 antibody (clone DO-7; monoclonal, DAKO, Carpinteria, CA, USA) diluted at 1 : 1500. This antibody reacts with both the wild-type and mutant type of the p53 protein. 

Immune cell markers included tumor-associated macrophages (CD68) and tumor-infiltrating lymphocyte subsets, such as CD3, CD4, CD8, and CD20, always evaluated in intraepithelial infiltrating cells located within cancer cell nests.

The advanced biotin-free polymer detection system was used (DAKO, Carpinteria, CA, USA). DAB (3.3-diaminobenzidine-tetrahydrochloride; Sigma, St. Louis, MA, USA) was applied as chromogen for five minutes, at room temperature. Sections were counterstained with hematoxylin. Positive and negative controls were run in the same batch of reaction. 

### 2.3. IHC Evaluation

**Figure 1:**
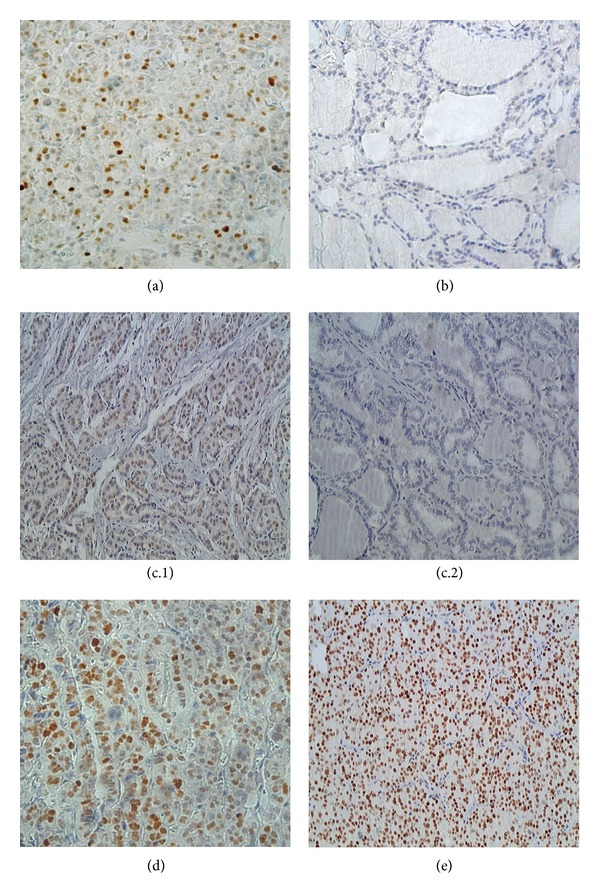
Immunohistochemical expression of p53 in thyroid lesions. Scarce cells are positive in one case of adenoma (×500); (b) negative goiter nuclei (×400); (c.1) papillary carcinoma showing high expression of p53 (×400); (c.2) papillary carcinoma showing negative expression; (d) follicular variant of papillary thyroid cancer showing moderate expression of the protein (×640); and (e) follicular carcinoma showing high expression of p53 (×400).

Slides were quantified by at least two of the authors (MAM, LLC and/or ECM) and then submitted to other two independent score evaluations performed by two experienced pathologists (JV and FAS), both blinded to tumor features. Cells were considered positive for p53 when a clear-cut brown staining was observed in the nucleus, as demonstrated in Figure 1. Tumor-infiltrating lymphocytes and tumor-associated macrophages were considered positive for immunohistochemical markers when a clear-cut brown staining was observed in the typical corresponding cellular localization. An individual evaluation of immune cell markers was completed for each tissue spot, estimating the number of positive cells per TMA spot, considering an approximate area of 0.79 mm^2^. The cases were grouped into categories for statistical analysis: negative (no positive cell) and positive (presence of positive cells in each spot).

Immunohistochemical expression of p53 was analyzed using the automated cellular imaging system ACIS-III (Chroma Vision Medical Systems, Inc., DAKO). Briefly, each tissue spot was digitalized through the system software, and a numerical value proportional to the percentage of stained nuclei was attributed by the computer analysis, as previously reported by our group [18].

### 2.4. *TP53 * Gene Sequencing and Analysis

The frozen tissues removed from the AC Camargo Hospital Biobank were subjected to histological analysis by pathologists (FAS and JV) to assess the percentage of malignant tumor tissue. Only samples with at least 70% of malignant cells were included in the study. The samples were manually dissected, and areas containing nonneoplastic tissues, fibrosis, fat, or other contaminants were removed. Genomic DNA from 78 tumor samples was extracted from frozen specimens, using a standard proteinase K-phenol-chloroform protocol. Exons 2–11 of *TP53 * (NM_000546) were directly sequenced. Each exon was individually amplified using 50 ng of genomic DNA. The polymerase chain reactions (PCRs) contained 0.2 *μ*mol/L of each primer, 0.2 mmol/L of each dNTP, 2.0 mmol/L MgCl_2_, and 0.5 U Platinum Taq DNA Polymerase (Invitrogen, Carlsbad, CA) in a final reaction volume of 20 *μ*L. The thermal cycling conditions included an initial cycle of 2 min at 94°C, followed by 35 cycles of 30 s at 94°C, 30 s at 57°C, 45 s at 68°C at specific primer annealing temperature of 57 or 59°C and a final extension of 10 min at 68°C. PCR products were purified with ExoSAP-IT (USB Products, Cleveland, OH) and sequenced in both directions. Sequencing reactions were run using Big Dye v.3.1 (Applied Biosystems, Carlsbad, CA) and separated on an ABI Prism 3130xl (Applied Biosystems). All sequences were analyzed using CLC Bio software (CLC Bio, Cambridge, MA) and compared with the *TP53 *sequence. All identified mutations were compared with *TP53 *in the International Agency for Research on Cancer database (http://p53.iarc.fr/). All pathogenic changes were confirmed in a second PCR reaction, using Platinum Taq High-Fidelity DNA Polymerase (Invitrogen) under the same conditions as above.

### 2.5. Statistical Analysis

Statistical analysis was carried out using the SAS System for Windows (Statistical Analysis System, version 9.1.3, Service Pack 3; Institute Inc, 2002-2003, Cary, NC, USA). Factors that affected recurrence-free survival were calculated using proportional hazard regression analysis and Kaplan-Meier survival curves with log-rank comparison. Nonparametric analysis of genotypes was performed using either the chi-square or Fisher's exact test, as indicated. A multivariate logistic regression model was applied using quantitative data of p53 immunostaining as dependent variables and clinicopathological features of tumor aggressiveness as explicative variables. Cox regression model was used to find independent prognostic markers. Mann-Whitney tests were used to compare continuous or arranged measures between two groups; Kruskal-Wallis test was used to compare three or more groups. The best cut-off point to predict malignancy was evaluated using receiver operating curve (ROC) analysis based on predicted probabilities from logistic regression models. The accuracy, sensitivity, specificity, and predictive values were also evaluated. All tests were conducted at the significance level *P* = 0.05.

## 3. Results

As expected, DTC patients were predominantly female (80.4%) aged 7–88 years old or 45.6 ± 16.3 years at the time of diagnosis.

### 3.1. P53 Protein Quantitative Analysis

**Figure 2:**
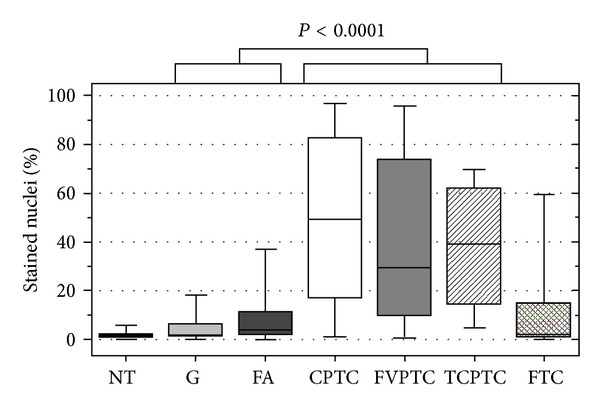
Box plot graph with the percentage of stained nuclei quantified by using ACIS III in all the studied thyroid tissues.

We defined p53 positivity when the tumor area presented at least 8.21% of nuclei stained. This cut-off value was based on a ROC curve, comparing malignant lesions (visually positive stained) with benignant lesions (predominantly negative). Among the 206 DTC patients studied, 127 (77.4%) out of 164 PTCs were positive for p53, and 11 (26.2%) out of 42 FTC were positive. When we considered PTC variants, 83 CPTC (79.0%), 41 FVPTC (75.9%), and 3 TCVPTC (75.0%) were positive for p53. The benign lesions presented less p53 positivity, given that only 27 (25.7%) out of 105 benign lesions presented some positivity for p53. FA were the benign lesions more frequently positive for p53: 19 out of the 55 adenomas (34.5%) presented positive nuclei. Among the goiters, positivity was even less frequent: only 8 out of the 50 lesions (16.0%) stained positive. No NT (0%) was considered positive for p53 staining (Figure 2). 

### 3.2. P53 for Diagnostic Purposes

The IHC quantitative analysis results, according to histopathological diagnosis, are summarized in Table 1 and demonstrate different expression levels among distinct thyroid tissues. P53 expression levels were significantly higher in malignant than in benign lesions (*P* < 0.0001). Using a cutoff of 8.210, a ROC curve distinguished malignant from benign cases with 71.7% sensitivity; 85.1% specificity; 93.6% positive predictive value; 49.5% negative predictive value; and 75% accuracy. A comparison of the different histological groups (NT, G, FA, FTC, CPTC, FVPTC, TCVPTC) showed that quantitative IHC analysis could differentiate NT from CPTC (*P* < 0.001); NT from FVPTC (*P* < 0.001); G from CPTC (*P* < 0.001); G from FVPTC (*P* < 0.001); FA from CPTC (*P* < 0.001); FA from FVPTC (*P* < 0.001); CPTC from FTC (<0.001); FVPTC from FTC (*P* < 0.001). Considering that the differential diagnosis of FVPTC from FTC and FA can be problematic, we investigated the utility of p53 as a diagnostic marker. A ROC curve identified 13.710 as a cutoff point that distinguished FVPTC from FA with 68.6% sensitivity; 81.5% specificity; 77.8% positive predictive value; 73.3% negative predictive value, and 75.23% accuracy. Using 6.740 as a cut-off point, we were able to differentiate FVPTC from FTC with 70.7% sensitivity; 78.4% specificity; 72.5% positive predictive value; 78.9% negative predictive value, and 74.9% accuracy. A box plot graph presented in Figure 2 demonstrates that although the different groups of lesions present considerable superposition of values, p53 expression levels were able to characterize them.

### 3.3. P53 as a Prognostic Marker

Since p53 profiles were significantly different among PTCs and FTC and protein expression was considerably higher and more frequent in PTC patients, who were the most prevalent in our cohort, we decided to further characterize p53 IHC profile compared with demographic characteristics, clinical pathological parameters, and outcome of PTC patients positive for p53 staining. These data are summarized in Table 2. 

Using tumor size categories (<2 cm, 2–4 cm, >4 cm), we observed that p53 was more frequently found staining smaller tumors (<2 cm) than tumors larger than 4 cm (*P* < 0.01). P53 was more frequent in solitary nodules than in multifocal tumors (*P* = 0.0286) and tended to appear more frequently in encapsulated tumors than in those without capsule, although the statistical comparison showed a marginal *P* value (*P* = 0.0762). In addition, p53 immunostaining was more frequent in patients without thyroiditis than in patients with thyroiditis (*P* = 0.0486), as presented in Table 2. Patients who presented metastasis at the diagnosis had fewer p53 stained nuclei than patients without metastasis (*P* = 0.0201). Unfortunately, the Kaplan-Meier survival curve was not able to confirm p53 as a factor affecting disease-free survival.

### 3.4. *TP53 * Genotypic Alterations

We investigated 78 PTC out of the 164 cases (54 CPTC, 23 FVPTC, and 01 TCVPTC) for the presence of genotypic alterations that could be related to protein expression and clinicopathological features. Sequencing data are summarized in Table 3. A comparison between p53 protein profile and the corresponding cases of genotype did not demonstrate any association, as shown in Table 4. Genotypes did not correlate neither with clinical nor with any pathological feature or with patient outcomes.

### 3.5. P53 Expression and Tumor-Associated Macrophages and Tumor-Infiltrating Lymphocytes

We further analyzed whether P53 expression would be associated with the presence of infiltration of immune cells. We observed that 89.6% of P53 positive tumors presented TAM, whereas only 71.4% of P53 negative tumors presented TAM (*P* = 0.002). We did not find any association between P53 expression and infiltration of CD3+ TIL. A marginal association was found between P53 expression and infiltration of CD4+ TIL (*P* = 0.051). Interestingly, we observed that 61.7% of P53 positive tumors presented infiltration of CD8+ TIL. However, CD8+ TIL were found in only 25.6% of P53 negative tumors (*P* < 0.001).

## 4. Discussion

Examining both p53 and *TP53 *gene in a clinical context, we demonstrated that protein profile may discriminate different groups of follicular thyroid lesions, confirming recent data that suggest that p53 may have a clinical utility as a diagnostic marker [19]. In addition, we found p53 expression to be associated with several characteristics well recognized as better prognosis factors such as tumor size, presence of a tumor capsule, presence of thyroiditis, absence of multifocal tumors, and absence of metastasis at the time of diagnosis [20, 21]. 

Somatic *TP53 *gene mutations have been reported in about half of all cancers and are believed to be critical determinants of the phenotype of many forms, including thyroid tumors [22]. *TP53* has long been known to be rarely mutated in well-differentiated thyroid tumors, whereas it is frequently altered in poorly differentiated or undifferentiated cancers [19, 23, 24]. However, the paradigm that this tumor suppressor gene is only involved in advanced cancer progression is contradicted by a series of evidence indicating that it also plays an important role in the early stages of gastric [25], hepatocellular [26], lung [27], and many other tumors, including thyroid cancer [23]. In fact, increased p53 protein levels were observed by immunohistochemistry not only in anaplastic and poorly differentiated thyroid cancer where p53 mutations are frequent but also in well-differentiated cancers, in the absence of any p53 mutation [28]. Confirming our data, Balta et al. recently showed that p53 was significantly augmented in PTC patients when compared with benign thyroid disorders [23]. These authors also found p53 more frequently expressed in tumors with more aggressive features, a finding difficult to interpret, since the patients they investigated did not correspond to the usual profile of DTC patients seen in clinical practice [29]. Conversely, other studies also found the protein associated with features of aggressiveness [30, 31], whereas some authors failed to find any relationship between p53 expression and clinicopathological features [24]. Differences concerning aggressiveness criteria and outcome characterization, in addition to the use of different p53 antibodies and IHC techniques, may explain these controversial results [29]. Our data, using a quantitative IHC analysis of a large number of patients managed according to a same protocol, and an antibody that recognizes both wild-type and mutant protein, indicate that p53 exerts an important physiological role in well-differentiated thyroid tumor containment, especially among papillary thyroid carcinomas.

We observed that tumors positive for p53 expression frequently presented an infiltration of a mixture of immune cells, such as CD8+ TIL and TAM. We previously demonstrated that the presence of CD8+ TIL and TAM is associated with nonaggressive phenotype of DTC [32]. The association of p53 expression and the presence of CD8+ TIL/TAM may help to explain, at least in part, the association between p53 expression and nonaggressive phenotype of our samples. 

It has been proposed that functional p53 protein is involved in the induction of antitumor CD4+ cytotoxic-T-cell activity against carcinoma cells [33]. In addition, p53 peptides may be processed and presented to host immune cells by a variety of mechanisms [34, 35]. In fact, the aberrant expression of p53 protein in tumor cells versus the normal expression in nontumor cells provides an immunological window for the use of p53 as a tumor antigen for immunotherapy [36].

In addition, we demonstrated a lack of relationship between p53 protein expression and *TP53* mutation status. Five out of the eight alterations we found are intronic and, therefore, may not result in protein alterations. Concerning the other three exonic alterations, one is a synonym mutation that probably does affect neither the gene nor the protein expression, which could explain why the corresponding levels of p53 protein were not augmented. Another is one of the most common *TP53 * polymorphisms; 26 out of 78 cases presented this polymorphism, and heterozygosis was found in 19 (73.08%) of the 26 cases presenting this exon 4 alteration. These last cases also did not present significant protein expression variation, suggesting that the presence of a wild-type allele could compensate the loss of function of the altered one, likewise described in many diseases in which carriers of only one mutant allele present a normal phenotype [37]. And the mutation in exon 10 has not been previously described in thyroid nodules. Our data do not support the current idea that p53 expression is directly related to the presence of mutations in TP53 gene [8, 38]. Conversely, they reinforce some recent results obtained in melanomas in which, despite the high levels of protein expressed, the frequency of mutations/polymorphisms in *TP53* gene was not high [39], suggesting that other mechanisms acting on the protein and/or its signaling pathway, such as p53 cytoplasmic retention [40] and MDM2 overexpression [41, 42], might be involved in wild-type p53 inactivation [38].

In conclusion, we suggest that p53 protein profile analysis by IHC might be useful in the differential diagnosis of thyroid lesions and may help characterize less aggressive cases that do not need hard-line management.

## Figures and Tables

**Table 1:** Immunohistochemical (IHC) expression of p53 protein using automated cellular imaging system ACIS-III quantitative analysis, according to histopathological diagnosis.

	Histopathology	Total number of patients	IHQ quantitative analysis in the whole group		Number of patients positive for p53	IHQ quantitative analysis for positive cases	
			Mean value	Median value		Mean value	Median value
Benign lesions	*Normal thyroid tissue *	16	1.76	1.11	0	—	—
	*Nodular goiter *	50	4.82	1.71	8	17.25	15.52
	*Follicular adenoma *	55	8.75	3.68	19	20.47	15.72
	*Papillary thyroid carcinoma *	164	51.85	53.59	127	52.32	63.69

Malignant lesions	Classic	106	42.16	32.24	83	62.17	70.70
	Follicular variant	54	38.15	39.18	41	54.30	57.92
	Tall cell variant	04			03	49.31	55.08
	*Follicular carcinoma *	42	11.11	2.17	11	36.36	21.64

**Table 2:** Immunohistochemical expression of p53 protein identified as positive, using automated cellular imaging system ACIS-III quantitative analysis, according to clinicopathological features of aggressiveness and patient outcome.

Clinicopathological features (*N*)	Quantitative analysis of p53 protein		*P*-value
	Mean	Median	
Gender (119)			
Female (101)	61.92	70.70	0.2940
Male (18)	53.96	53.35	
Ethnicity (115)			
White (111)	77.59	83.42	0.5473
Non-White (4)	78.70	78.70	
Age (119)			
≤45 years old (65)	60.29	69.78	0.8791
>45 years old (54)	61.22	64.03	
Smoking habit (65)			
Smoker (24)	73.65	78.89	0.3998
Nonsmoker (39)	78.43	84.84	
Tumor size (119)			
<2 cm (63)	69.54	77.91	**0.0015**
2–4 cm (35)	55.80	56.93	
>4 cm (21)	42.40	32.72	
Extrathyroidal invasion (117)			
Yes (60)	57.26	57.12	0.0969
No (57)	65.73	78.72	
Capsulation (102)			
Yes (30)	65.53	71.74	0.0762
No (72)	54.47	53.45	
Multifocality (119)			
Yes (45)	53.27	55.08	**0.0286**
No (74)	65.24	74.14	
Metastasis at diagnosis (119)			
Present (43)	51.34	46.01	**0.0201**
Absent (76)	66.02	75.75	
Thyroiditis (119)			
Present (39)	69.38	78.72	**0.0486**
Absent (80)	56.49	57.12	
Disease stage (TNM) (124)			
I (79)	62.23	70.70	0.6140
II (13)	58.32	74.44	
III (15)	51.35	53.59	
IV (1)	86.87	—	
IVa (11)	55.87	57.31	
IVb (4)	43.29	41.02	
IVc (1)	56.93	—	
Outcome (109)			
Free of disease (100)	58.26	60.14	0.9605
Recurrent (9)	57.14	57.31	

**Table 3:** *TP53* genetic alterations in PTC patients, according to histopathological subtypes and the mean p53 protein expression quantified with the automated cellular imaging system ACIS-III.

Histopathology of PTC (total of cases)	*TP53 *alteration							Expression of p53 protein	
	Number of cases	Distribution of alleles	Nucleotide	Local	AA change	Mutation type	dbSNP ID	Mean	Median
CPTC (54)	43	16 CG/27 GG	C11117G	Intron 2	—	Intronic	rs1642785	65.70	77.22
	18	14 GC/04 CC	G11446C	Exon 4	R72P	Missense	rs1042522	62.48	74.40
	02	02 AG	A12708G	Exon 6	R213R	Silent	rs1800372	74.88	74.88
	04	04 CT	C13491T	Intron 7	—	Intronic	rs12947788	88.45	90.89
	01	01 AT	A17021T	Intron 10	—	Intronic	rs17880847	Unavailable tissue	
	01	01 TG	T16987G	Éxon 10	S366A	Missense	rs17881470	94.80	—

FVPTC (23)	18	07 CG/11 GG	C11117G	Intron 2	—	Intronic	rs1642785	71.54	85.04
	01	01 CA	C11299A	Intron 3	—	Intronic	rs17883323	92.61	—
	08	05 GC/03 CC	G11446C	Exon 4	R72P	Missense	rs1042522	77.49	94.45
	01	01 AG	A12708G	Exon 6	R213R	Silent	rs1800372	59.59	—
	02	02 CT	C13491T	Intron 7	—	Intronic	rs12947788	52.71	52.71
	01	01 GA	G13925A	Intron 8	—	Intronic	—	95.66	—

TCVPTC (1)	01	01 GG	C11117G	Intron 2	—	Intronic	rs1642785	23,28	—

**Table 4:** Comparison between p53 IHC profile and corresponding *TP53 *genotype.

	Altered cases		Wild-type cases		*P* value
	Mean	Median	Mean	Median	
Intron 2	73.35	71.79	83.42	77.99	N.S.
Exon 4	66.68	77.10	76.20	83.42	N.S.
Exon 6	69.78	70.70	73.15	82.83	N.S.
Intron 7	72.70	81.39	76.55	90.72	N.S.
Intron 3	92.61	∗	73.59	82.15	∗
Intron 10	Tissue unavailable for IHC analysis		73.61	82.15	—
Exon 10	94.80	∗	73.74	83.43	∗
Intron 8	95.66	∗	77.34	83.43	∗

*Analysis was not possible because only one patient had alterations.
